# Strategies to improve palatability and increase consumption intentions for *Momordica charantia *(bitter melon): A vegetable commonly used for diabetes management

**DOI:** 10.1186/1475-2891-10-78

**Published:** 2011-07-28

**Authors:** Laura S Snee, Vivek R Nerurkar, Dian A Dooley, Jimmy T Efird, Anne C Shovic, Pratibha V Nerurkar

**Affiliations:** 1 Department of Human Nutrition, Food and Animal Sciences (HNFAS), College of Tropical Agriculture and Human Resources (CTAHR), University of Hawaii, Honolulu, HI, USA; 2 Department of Tropical Medicine, Medical Microbiology and Pharmacology, John A. Burns School of Medicine, University of Hawaii, Honolulu, HI, USA; 3 Center for Health Disparities Research and Department of Public Health, Brody School of Medicine, Greenville, NC, USA; 4 Laboratory of Metabolic Disorders and Alternative Medicine, Department of Molecular Biosciences and Bioengineering (MBBE), CTAHR, University of Hawaii, Honolulu, HI, USA

## Abstract

**Background:**

Although beneficial to health, dietary phytonutrients are bitter, acid and/or astringent in taste and therefore reduce consumer choice and acceptance during food selection. *Momordica charantia*, commonly known as bitter melon has been traditionally used in Ayurvedic and Chinese medicine to treat diabetes and its complications. The aim of this study was to develop bitter melon-containing recipes and test their palatability and acceptability in healthy individuals for future clinical studies.

**Methods:**

A cross-sectional sensory evaluation of bitter melon-containing ethnic recipes was conducted among 50 healthy individuals. The primary endpoints assessed in this analysis were current consumption information and future intentions to consume bitter melon, before and after provision of attribute- and health-specific information. A convenience sample of 50, self-reported non-diabetic adults were recruited from the University of Hawaii. Sensory evaluations were compared using two-way ANOVA, while differences in stage of change (SOC) before and after receiving health information were analyzed by Chi-square (χ^2^) analyses.

**Results:**

Our studies indicate that tomato-based recipes were acceptable to most of the participants and readily acceptable, as compared with recipes containing spices such as curry powder. Health information did not have a significant effect on willingness to consume bitter melon, but positively affected the classification of SOC.

**Conclusions:**

This study suggests that incorporating bitter foods in commonly consumed food dishes can mask bitter taste of bitter melon. Furthermore, providing positive health information can elicit a change in the intent to consume bitter melon-containing dishes despite mixed palatability results.

## Background

Prevalence of obesity and associated metabolic syndromes, such as type 2 diabetes mellitus (T2DM) and cardiovascular diseases (CVD) are escalating worldwide. In the United States, an estimated 20.8 million people have T2DM with an estimated two to four-fold increased risk of CVD [1]. Treatment of T2DM usually requires multiple interventions such as exercise, diet modification and pharmacotherapy [2,3]. Anti-diabetic drugs are prone to adverse events and may not have any direct effect on the impaired plasma lipid profile, requiring multiple drug therapy [4]. Furthermore, recent surveys indicate that regardless of ethnicity, about 80% of diabetic individuals use complementary and alternative medicine therapies [5]. Overall, there remains a need for effective therapeutic approaches that will not only normalize blood glucose and improve insulin sensitivity, but also improve plasma lipid profile.

*Momordica charantia *(commonly known as bitter melon) has been widely used throughout the world for centuries, to manage diabetes and its complications [6]. It is a member of the *Cucurbitaceae *family of vegetables and often consumed in South America, Asia, Africa, the Amazon, and the Caribbean. Evidence from animal and *in vitro *studies indicates that bitter melon not only improves glucose metabolism, but also ameliorates obesity and associated diabetic dyslipidemia [7-11]. A few clinical trials have been conducted, many of which were flawed by small sample sizes, lack of proper randomization or placebo control groups [12-15]. A recent review by Leung et al. [16] has elegantly summarized and reviewed clinical trials conducted until 2009 to test the hypoglycemic effects of bitter melon.

One of the most recent studies by Fuangchan et al. [17] effectively demonstrated the hypoglycemic effect of bitter melon among type 2 diabetic individuals receiving 2,000 mg/day of dried bitter melon powder. This was a multicenter, randomized, double blinded trial for four weeks that further demonstrated a significant reduction in fructoseamine levels among diabetic patients consuming bitter melon [17].

Medicinal value of bitter melon has been attributed to its high antioxidant properties due in part to phenols, flavonoids, isoflavones, terpenes, anthroquinones, and glucosinolates, all of which confer a bitter taste [18]. Removal of active bitter components, through a variety of debittering processes as well as selective breeding can result in loss of possible health benefits. Our long-term goal is to test the efficacy of bitter melon as a preventive, complementary and/or alternative treatment for diabetes and its complications. However, the feasibility of clinical trials using bitter melon may be limited due to its unacceptable bitter taste. Therefore, the primary aim of the study was to develop recipes that would mask the bitter taste of bitter melon and improve its palatability. Furthermore, to increase acceptance and consumption intentions in general population, we incorporated bitter melon into food dishes that are popular and routinely consumed among the various ethnic groups in Hawaii.

Although palatability is indicated to be more important than health information when making individual food choices [19], few studies have investigated the effects of repeated exposure and health information on acceptance of various foods [20,21]. Therefore, the second goal of this study was to determine the effect of bitter melon-associated nutrition and health information on willingness or intent to consume bitter melon-containing food dishes.

## Methods

### Recipe development and preparation

Chinese variety of bitter melon is available in Hawaii throughout the year and was obtained from a local grocery store. Bitter melon is used in numerous traditional Asian cuisines, a popular being whole bitter melon stuffed with meats. However, most of them are bitter to taste, curtailing its wide-spread therapeutic use. Therefore, initial recipe development was based on a pilot study where a small convenience group (7 females and 1 male, n = 8; 5 Caucasians, 1 Pacific Islander and 1 Indian) were asked to chose their favorite food dishes into which bitter melon was incorporated (chili, tomato sauce, curry, chicken stir fry, and hummus). The same individuals tasted these bitter melon-containing food dishes and completed sensory evaluations based on a 9-point hedonic scale (1 = dislike extremely, 2 = dislike very much, 3 = dislike moderately, 4 = dislike slightly, 5 = neither like nor dislike, 6 = like slightly, 7 = like moderately, 8 = like very much and 9 = like extremely), initially developed by Peryam and Pilgrim [22]. The hedonic scale balanced with respect to degrees of liking, is often used to determine consumer acceptability. The scale can be used by all individuals even if they are untrained in sensory evaluations, thus making it ideal for studying a representative sample within a specific population [23]. This scale was used to evaluate bitter melon recipe attributes for bitterness, smell, overall liking and taste, which were deemed important to bitter melon acceptability. Overall, our preliminary data indicated that tomato-containing dishes such as chili and pasta sauce were able to mask the bitter taste efficiently when compared with spices in curry, soy sauce in the stir-fry dishes or garlic in humus (hedonic score, 5.1 ± 3.2).

**Figure 1:**
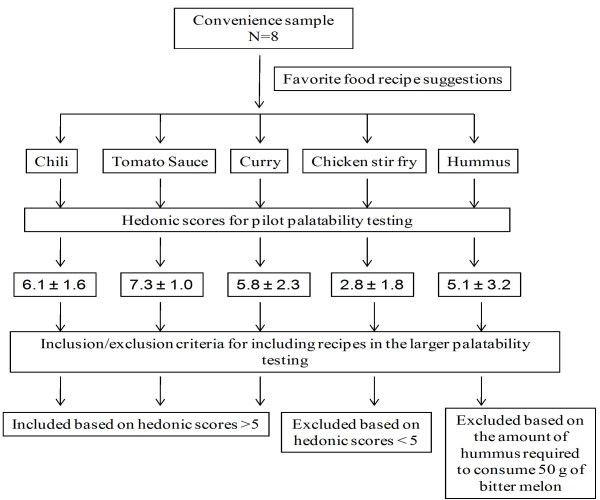
**Pilot study outline and hedonic scores of bitter melon-containing food dishes for development of recipes**.

The flow chart (Figure 1) outlines the rationale for using vegetarian chili, curry, and pasta sauce in our subsequent larger study, which was based on hedonic score cut-off value of 5.0 as well as their ease of preparation, and cost. These food dishes included ingredients, such as tomato and spices, which were intended to mask the bitterness of the bitter melon. Ingredients in our recipes such as tomato, garlic and spices can also lower blood glucose and lipids levels as indicated by studies conducted in rodents and humans [24-26]. The stir fry bitter melon was deemed unpalatable by the group as evidenced by low acceptance hedonic score. All but one individual indicated on the survey form that he/she would not eat stir fry bitter melon again in the future. Furthermore, since the amount of hummus required to consume 50 g of bitter melon would have been unrealistic in a clinical trial it was excluded from the larger palatability testing study.

Minestrone soup contains a tomato base and is similar to chili and pasta sauce in taste and ingredients, but is different in texture due to diced bitter melon versus puréed bitter melon. It was therefore included in the larger study to increase the number of food choices and replace the bitter melon-containing stir fry dish. In the larger study, bitter melon was added to basic recipes of chili, curry, minestrone soup, and tomato sauce for pasta, that are commonly consumed food dishes by one or more ethnic groups in Hawaii. Each dish contained 50 g of bitter melon per one cup (250 mL) serving. Current clinical studies have used up to 50 to 60 mL of bitter melon juice per day, divided in two to three doses [27]. Ayurveda recommends about 2-3 tsp of bitter melon juice for a diabetic adult, twice a day [28]. Approximately 30 mL of juice was obtained from 50 g of bitter melon fruit without seeds and pulp. Although clinical trials using uncooked, dried powder up to 2,000 mg/day have demonstrated a hypoglycemic effect of bitter melon, these effects were not equivalent to metformin effects [17]. We therefore developed recipes with 50 g of bitter melon per serving of one cup.

Foods were prepared seven days prior to the sensory evaluation, cooled to room temperature and frozen at -20°C. This was done in anticipation of future clinical trials that likely will require the foods to be frozen for several days in order to facilitate distribution and quality control. On the previous day of the sensory evaluations, foods were placed in the refrigerator to thaw. On the morning of the sensory evaluation, food dishes were reheated to 70-73°C, kept warm at 60°C using electric crockery pots and served warm throughout the study. In Hawaii, it is popular to consume chili or curry with rice, while pasta sauce is traditionally used with pasta. Since both the starches are bland in taste, their contribution towards the taste of bitter melon-containing dishes is expected to be negligible. Therefore the chili and curry dishes were served with white rice while tomato sauce was served with pasta, as customarily served in Hawaii. A randomized block design was followed to specify the order of serving food dishes.

### Study participants

This study was reviewed and approved by the University of Hawaii's Institutional Review Board Committee on Human Studies. A quasi-random, point of entry sample of 50, self-reported non-diabetic adults were recruited from the University of Hawaii Manoa campus, using paper and electronic flyers, to participate in a cross-sectional sensory evaluation of bitter melon-containing food dishes. Exclusion criteria included self-reporting individuals with hypoglycemia, diabetes, any other chronic illness requiring medications and those with allergies/sensitivities to study food components. Additionally, women who were pregnant, breastfeeding, or thought they might be pregnant as well as children were excluded due to safety concerns [6]. A written informed consent was obtained from all the study participants.

Consumption of uncooked bitter melon powder or juice in larger quantities up to 2,000 mg/day or 2,000 mL and for longer durations may cause adverse events such as stomach pain, cramps or diarrhea [16,17]. Being a palatability testing study, the total amount of cooked bitter melon consumed was less than 25 g. Although participants were informed of the possible adverse events, they were not followed up. Participants did not self report any adverse events within 72 h of palatability testing.

### Survey part I

The sensory evaluation forms consisted of two parts. The first part included participant demographics and sensory evaluation questions for each food dish. Participants were randomly assigned to one of the four sensory evaluation stations, based on the order in which they arrived for the study. Each station had one of the four food dishes containing bitter melon. After initial sampling, each participant proceeded to the next station such that the order was always the same (chili, tomato sauce, curry, soup). Each participant was given a sensory evaluation form along with approximately 1-2 tablespoons of each food dish served over 1 tbsp (approximately 15 g) of rice or pasta in the order they were to complete the sensory evaluation. Participants were asked to taste each of the above food samples one at a time in the order on the form. Water and saltine crackers were provided for participants to cleanse their palates between sampling as a measure to offset "cross-over effect" bias. Participants evaluated each dish by considering how much they liked or disliked an individual dish, as well as the overall characteristics, and indicating their opinion by placing a [√] in one box only. Participants were requested to refrain from discussing their opinions with others and compliance was monitored by study coordinators. During data analysis, numerical scores were generated by authors based on the above mentioned hedonic scale. Participants also listed factors that would improve acceptability and increase consumption frequency for each dish. Sensory evaluations took approximately 10 minutes to complete. Upon completion, participants answered questions related to their current consumption, knowledge, and attitudes, as well as to their intentions to consume bitter melon dishes as a meal in the future, over a period of two weeks.

### Survey part II

The second part of the survey form was designed to determine previous bitter melon consumption as well as future intentions to consume bitter melon-containing dishes after receiving bitter melon-associated health information. Initially, individuals were placed in one of the Stages of Change (SOC) applicable to dietary studies based on their answers to questions provided in an algorithm format as shown in Table 1[29]. The model is based on six stages: *precontemplation*, defined as a stage when an individual has no intentions of changing behavior within six months and remains resistant to change, *contemplation*, defines an individual who has limited awareness of potential problems or risks and contemplates making changes, *preparation*, defines an individual who is planning a change through specific action within the next month, *action*, defines an individual who conducts the specific behavior, *maintenance*, classified as the point in which the behavior has been occurring for at least six months and *termination*, the point at which a previous behavior is completely eliminated and is no longer a possibility when temptation arises. In the last part of the survey, participants received "attribute-specific" and "consequence-specific" health information in writing as described by Wansink, *et al*.,[30] and adapted for this study.

**Table 1:** Bitter Melon Stages of Change Algorithm*

Question	Answer	Stage
Do you currently consume bitter melon at least once every 2 months?	Yes	Action or Maintenance
	
	No	Pre-action

Which of the following best describes how long you have consumed bitter melon at least once every 2 months?	<6 months	Action
	
	≥ 6 months	Maintenance

I am not thinking of consuming bitter melon in the future.	Yes	Precontemplation

I am thinking of consuming bitter melon in the future.	Yes	Contemplation

I am planning to consume bitter melon in the future.	Yes	Preparation

*Adapted from Chapman-Novakofski [29]

Knowledge about bitter melon traits or characteristics of action was provided by attribute-specific information. For example, "research with animals and humans have shown that bitter melon can lower blood sugar levels" provides information about bitter melon attribute or quality. The consequences of such attributes were provided separately. An example of consequence-specific information included in the survey was "eating bitter melon may help lower your risk for type 2 diabetes or heart disease". Surveys were randomized such that half of the participants read the attribute-specific information first while the other half read the consequence-specific information first.

After receiving each set of information, participants were asked to again list how many times they would be willing to consume each dish as a part of meal over a two-week period. They were asked to check only one box for each sample that included zero times, one time, two times, three times, four times or five times per week. This was designed to assess changes in willingness to consume each dish given varying levels of knowledge about bitter melon. Additionally, individuals initially classified in the pre-action stages (precontemplation, contemplation, preparation) were asked after each set of information to check one of three boxes to indicate that they were either "not thinking," "thinking," or "planning" on consuming bitter melon in the future. This was included to determine if providing health information affected stage movement for these participants despite palatability of bitter melon. SOC classification was reevaluated for individuals in the pre-action stages after each set of health information to determine if the information had an effect on stage movement.

### Statistical analyses

Survey data were analyzed using SAS (version 9, Cary, N.C.) and GraphPad Prism, Prism 5 for Windows, version 5.01. Frequency distributions among the hedonic scale categories were determined for each of the three sample attributes (texture, bitterness, and smell) as well as the overall sample rating. Statistically significant differences for each attribute were assessed using a two-way ANOVA. *Post hoc *pair-wise multiple comparisons were evaluated using the Bonferroni test after ANOVA. Similarly, a two-way ANOVA model with Bonferroni post test was used to determine if participant consumption intentions for each sample were affected by participants receiving "attribute-specific," "consequence-specific," or both types of information about bitter melon compared with those receiving no information. Chi-square (χ^2^) analyses were conducted to determine if there were statistically significant differences in participants' stage distribution before and after receiving health information as well as the order of providing information. Results were considered significant at p < 0·05.

## Results

### Participant demographics

All 50 participants completed all or part of the sensory evaluation and survey. One participant survey was excluded from the analysis due to unreliable demographic responses. Table 2 demonstrates the demographic characteristics for the study participants (n = 49). There was nearly equal distribution of female (53%) and male (47%) participants. Most participants (47%) identified with one of the nine major Asian ethnic or racial groups in Hawaii, specifically Chinese (18%) or Japanese (14%), while 41% identified themselves as "White". One of the 49 participants included in the analysis did not provide their age. Of the participants that did provide their age (n = 48), the mean age was 31.8 + 12.5 (SD).

**Table 2:** Demographic characteristics of study participants (n = 49)

Characteristic	n	%^1^
**Gender**		

Male	23	47%

Female	26	53%

**Ethnicity**		

Caucasian (white)	20	41%

Chinese	9	18%

Japanese	7	14%

Hawaiian/Part-Hawaiian	3	6%

Filipino	2	4%

Indian (India)	2	4%

Korean	2	4%

Mexican	1	2%

Samoan	1	2%

Other	2	4%

**Age (yr)**^2^		

18-24	17	35%

25-29	13	27%

30-39	6	13%

40-49	5	10%

50-59	5	10%

60-65	2	4%

^1^Percentages may not add up to 100 due to rounding.

^2^Age provided by 48 participants

### Hedonic scores for bitter melon-containing recipes

Overall acceptability of specific attributes varied among the four food samples. Mean hedonic scores for attributes and overall liking are presented in Table 3. Mean hedonic scores greater than 5 (midpoint) were considered acceptable while those less than 5 were deemed unacceptable. Except for the curry, all samples were considered acceptable to some degree. The tomato sauce received the highest overall mean hedonic scores, followed by the chili, soup, and curry, respectively. However, overall mean hedonic scores for the soup and chili were not significantly different (p < 0.05). The curry received significantly lower mean hedonic scores for all attributes except smell, (Table 3). The bitterness of curry, chili and soup was considered unacceptable based on the hedonic scores (Table 3). Although there was a range of responses for each dish, it is evident that most people largely disliked the curry to some degree while most liked the tomato sauce which received the highest mean hedonic scores for all attributes, including bitterness (5.88 ± 2.0).

**Table 3:** Hedonic scores for bitter melon-containing food dishes

	Texture	Bitterness	Smell	Overall
*Food dishes consumed with rice*				

**Chili**	6.6 ± 1.6^a^	5.0 ± 2.2^a^	6.5 ± 1.7^a^	5.5 ± 2.8^a^

**Curry**	5.6 ± 1.7^c^	3.3 ± 2.2^c^	5.5 ± 2.1^c^	3.5 ± 2.1^c^

*Food dishes consumed with pasta*				

**Tomato Sauce**	7.0 ± 1.4^b^	5.9 ± 2.0^b^	7.2 ± 1.1^b^	6.7 ± 1.4^b^

				

**Soup**	6.2 ± 1.7^a^	5.1 ± 2.3^a^	6.3 ± 1.8^a^	5.4 ± 2.3^a^

Values depict scoring Scale: 1 = extremely dislike; 5 = neither like nor dislike; 9 = extremely like and are represented as mean ± SD

^a, b, c^Means within attributes, followed by the same superscript are not different (p < 0.05)

**Figure 2:**
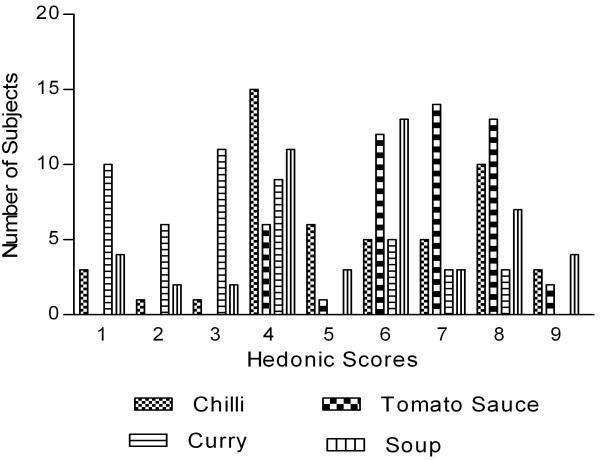
**Frequency distribution of hedonic scores for each food dish (n = 49)**. 50 g of bitter melon was incorporated into standard recipes of chili, tomato sauce, curry and soup. Data represents the hedonic score distribution: 1 = extremely dislike; 5 = neither like nor dislike; 9 = extremely like.

Figure 2 shows the frequency distribution among hedonic responses for the overall liking scores for each food dish. Responses for the chili and soup were mixed and more difficult to determine their acceptability from only distribution responses. Over 30% of participants indicated they "dislike slightly" the chili while another 20% said they liked it very much. Similarly, 23% of participants indicated they "dislike slightly" the soup, yet 27% said they liked it slightly. The remaining participant responses were near evenly split between the degrees of liking or were neutral as indicated by a "neither like nor dislike" response. Overall mean hedonic scores for the chili (5.51 ± 2.17) and soup (5.39 ± 2.26) were above a score of 5 and therefore considered acceptable (Table 3). The percentage of participants that typically did not like chili, curry, soup, or tomato sauce varied from 0% to 17% (Figure 2). The primary theme of comments on how to make each dish better was to decrease or somehow mask the bitter taste or aftertaste (Table 4). However, the distribution of scores for bitterness and the other attributes varied by food dish.

**Table 4:** Examples of comments on factors that would improvise acceptability and consumption frequency of bitter melon-containing food dishes.

Recipes	Comments to the question "How could this dish be better so that you would consider eating it?"
**Chili**	➢ "Use more beans, they help temper the bitterness; needs stronger flavor (try adding chipotle pepper)"
	➢ "I think this is a great way to incorporate bitter melon."
	➢ "Onions and garlic"
	➢ "Adding more sweetener"
	➢ "More rice"
	➢ "Add salt"
	➢ "Needs more spices; I like spicy chili"
	➢ "I think meat could overpower the bitterness"

**Tomato Sauce**	➢ "Less bitter, less hard chunks"
	➢ "Don't like bitter food; needs meat"
	➢ "It could be better with more sugar."
	➢ "This was very good! It would be very good with scallops, as they have a nice contrasting 'sweetness'."
	➢ "Maybe a little more spices"
	➢ "More garlic"
	➢ "I thought the flavor, texture, & seasoning were all pretty good. Maybe if some cheese were added it would make it more appealing."
	➢ "Still quite bitter, but the spices actually cover it well. The spiciness is quite strong."

**Curry**	➢ "Way too bitter; needs meat"
	➢ "It would be better with more gravy"
	➢ "Maybe add other veggies; it was really bitter."
	➢ "Could have more curry and spices"
	➢ "More salt and spice; too plain"
	➢ "Stronger curry spice; garlic would help; and spice peppers"
	➢ "More garlic"
	➢ "More sugar or I would eat it when I'm sick."

**Minestrone Soup**	➢ "Make it more sweet or salty to cover up the bitter after taste."
	➢ "This would actually be a really good dish with a little more garlic and salt."
	➢ "Less pepper taste; love the veggies (carrot & corn)"
	➢ "The spices really contribute a lot to this dish and bitter melon taste is minimal; maybe more spices or tomato flavoring?"
	➢ "Add meat"
	➢ "More herbs and seasoning"
	➢ "I liked that it was flavored well and that the bitter melon flavor was pretty well balanced. I wouldn't change it."
	➢ "Less bitter"
	➢ "It was too bitter; maybe use less bitter melon. More garlic might be good."

### Health information has no effect on consumption intentions

Table 5 shows the number of times (mean ± SD) participants indicated they would be willing to consume each food dish in a two-week period both before and after receiving health information about bitter melon. Consumption intentions were significantly lower for the curry than all other dishes both before and after providing health information (Table 5, p < 0.05). In contrast, consumption intentions were significantly higher for tomato sauce, before and after providing attribute specific information (Table 5, p < 0.05). Although there were no significant differences in the mean values for consumption intentions among the chili and soup, the actual distribution of responses varied among these dishes (data not shown). Prior to receiving health information, 65% of the participants were unwilling to eat curry, 25% of participants indicated that they would not be willing to consume either chili or soup while 14% of participants indicated that they were unwilling to eat the tomato sauce in a two-week period (data not shown). The percentage of participants unwilling to consume soup and chili dropped to 20 - 21% after receiving health information, but was statistically insignificant (data not shown). Overall, the consumption intentions were the highest for tomato sauce and lowest for curry regardless of the health information provided and significantly differed from other bitter melon-containing recipes tested (Table 5, p < 0.05)

**Table 5:** Number of times participants were willing to consume each dish in two weeks Pre- and post-health information.

	Chili	Tomato Sauce	Curry	Soup
**No Information**	1.6 ± 1.5^a^	1.9 ± 1.4^b^	0.6 ± 1.0^c^	1.6 ± 1.4^a^

**Post-Attribute Information**	1.9 ± 1.5^a^	2.2 ± 1.5^b^	0.7^b ^± 1.2^c^	1.7^a ^± 1.5^a^

**Post-Consequence Information**	1.9 ± 1.6^a^	2.1 ± 1.5^a, b^	0.7^b ^± 1.2^c^	1.8^a ^± 1.5^a^

Data is represented as mean ± SD

^a, b^Means within rows followed by the same superscript are not different (p < 0.05)

### Health information had limited effect on stage distribution

**Figure 3:**
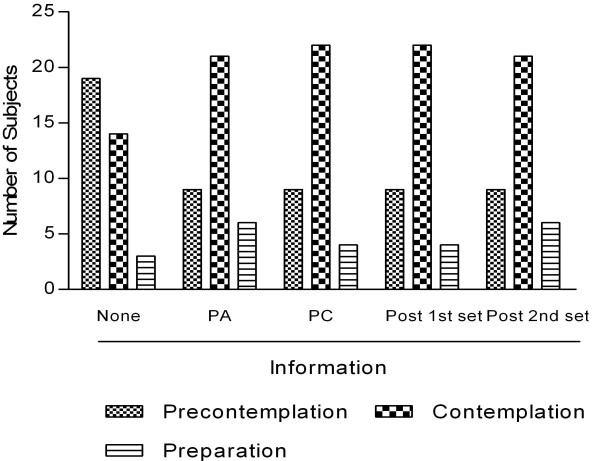
**Depicts the percentage of participants in pre-action stages after receiving "attribute- and consequence-specific" information (n = 49)**.

Of the 49 participants, 69% (n = 34) indicated they had tried bitter melon before, while 31% (n = 15) had never eaten bitter melon. However, only 13 of these 34 participants (38%) indicated that they previously consumed BM at least once every two months. Consumption at least once every 2 months was used as the means to classify an individual in the "action stages" according to the SOC algorithm shown in Table 6. Based on responses to the questions in Table 1, the stage distribution of participants was determined after they evaluated each sample, but prior to receiving health information about bitter melon. Table 6 shows the number of individuals classified in the pre-action and action stages for bitter melon consumption prior to receiving health information. A total of 13 participants classified in the action stages specified one or more reasons why they consumed bitter melon (I like the taste, n = 8; I grew up eating bitter melon, n = 9; health, n = 6 and other such as friends prepare food with bitter melon, n = 1). After receiving health information about bitter melon, stage distribution shifted for those participants initially classified in the pre-action stages. Figure 3 depicts the percentage of participants in the various pre-action stages before receiving information and after receiving either attribute-specific or consequence-specific information. There was no significant effect on stage distribution based on the order of attribute- or consequence -specific information (Table 6).

**Table 6:** Stage distribution pre-health information

Stages	n	n_total_	%_total n_	n_pre-action_	%_pre-action_	n_action_	%_action_
***Pre-action***	**36**	**49**	**74%**				

precontemplation	19	49	39%	36	53%		

contemplation	15	49	31%	36	42%		

preparation	2	49	4%	36	6%		

***Action ***	**13**	**49**	**27%**				

action	3	49	6%			13	23%

maintenance	10	49	20%			13	77%

N = number of participants in each stage of distribution, n_total _= number of total participants in the study, %_total n _= percent of total number of participants in each state distribution, n_pre-action _= number of participants in pre-action stage, %_pre-action _= percentage of pre-action participants, n_action _= number of action participants and %_action _= percentage of participants in each action or maintenance.

*precontemplation*, participants with no intentions of eating bitter melon, *contemplation*, participants with limited information about bitter melon and contemplates making changes, *preparation*, participants planning to change and consume bitter melon, *action*, participants who consume bitter melon, *maintenance*, participants who consumed bitter melon for at least 6 months

The number of individuals in the precontemplation stage significantly decreased by more than half from 53% to 25% and 26% after individuals received "attribute- and consequence-specific" information, respectively (p < 0.05, Figure 3). The number of participants increased in the contemplation and preparation stages. Stage distribution in the precontemplation and contemplation stages was significantly different after participants received health information (p < 0.05, Figure 3). Thirty-six percent (n = 13) of all participants initially classified in the pre-action stages shifted stage after receiving health information. Participants who started out in the preparation stage (n = 3) did not shift stages based on the questions used to assess stage change. Stage changes after participants received health information were mostly between the precontemplation and contemplation stages although some movement occurred between the contemplation and preparation stages. Movement across more than one stage was not observed in this study. These results are expected based on the nature of individual behaviors within each stage. If those participants are excluded, then 39% (n = 13) of the pre-action participants who were able to shift stage (n = 33) as indicated by their survey responses.

## Discussion

Traditionally, bitter melon has been used as a vegetable in a variety of ways in Asian cuisine, and as the main ingredient its bitter taste can be overwhelming. Bitter melon-containing food dishes are not popular in the Western world due to its bitterness, which is an acquired taste. We therefore conducted a pilot study to incorporate bitter melon into few popular food dishes regularly consumed in Hawaii.

Our results indicate that chili, soup, and tomato sauce were more palatable compared to the curry dish which was considered unacceptable due to its "bitterness". It is possible that a sampling bias could have existed solely by the use of the term "bitter melon" in the recruitment process. Participants were aware from signs used to advertise the study, that it would be a tasting or acceptability study of bitter melon. Additionally, one of the main attributes singled out for hedonic evaluation was "bitterness" which could have drawn more attention to this potentially negative food trait even before the tasting began. To date, there are no published palatability studies of food dishes prepared with bitter melon. In one related study, hedonic ratings for bitter melon "pleasantness" before exposure to a bittersweet beverage for seven days and post-exposure were 2.9 ± 0.2 and 3.2 ± 0.3 (mean ± SE), respectively [21]. These values were similar to the average "pleasantness" hedonic ratings of bitter melon and another bittersweet beverage of 3.2 ± 0.4 and 3.4 ± 0.5 for low- and high-exposure groups, respectively, reported by Mattes [20]. In our study, the mean hedonic curry scores for "bitterness" (3.27 ± 2.22) and "overall" liking (3.53 ± 2.14) for the curry dish were similar to those previously reported [20,21]. Furthermore, since 65% of the participants were between the ages of 24 to 35 years, it is highly possible that hedonic score about bitterness could be influenced by age, as older populations are more tolerant to bitter taste due to blunted gustatory sensations.

Although spices in the curry were unable to mask the bitter taste, its low total energy content makes it an attractive therapeutic alternative in Asian countries such as India wherein curry preparation may be culturally preferred and more acceptable. The other three dishes consisted of several other ingredients such as tomatoes, garlic and basil that masked their bitterness. Some traditional Asian recipes remove the bitter flavor from the fruit by adding salt, which extract the bitter juices and is then discarded. Alternatively, a combination of salt and vinegar can also be used to mask the bitter flavor. Besides salt, natural and artificial sweeteners as well as fat or cooking techniques such as pickling, caramelizing, or braising can be effective in reducing bitter taste [18,31,32]. In our study, increasing fat or sugar content to improve palatability was avoided since the dishes are intended for future clinical trials among diabetic individuals.

Since this was a palatability testing, the variation in calories of different food dishes is not expected to affect the taste. Similarly, bland starches such as rice and pasta are not expected to significantly affect palatability of bitter melon-containing food dishes. Although participants commented on "more rice" or "less rice" to increase palatability, there was no preference for one starch over the other. Moreover, these bitter melon-containing dishes could have been consumed without the starches. However, that may have resulted in a false positive or negative palatability score as these food dishes are generally consumed with starches.

Besides taste, health information also may influence the gradual acceptance and consumption of functional foods with bitter taste [33]. In our study, health information failed to have a significant effect on consumption intentions. This is in contrast to previous study indicating that likelihood of consumption is decreased as bitterness increased, while intentions increased with only certain information on health benefits [34]. Failure to see a change in consumption intentions could be a consequence of age since 35% of our participants were young (below 24 years) and healthy, and therefore may not have had the same health concerns as older populations. Earlier studies by Caltabiano and Shellshear indicated that health was less important in young adults (mean age of 22 years) than taste or palatability when determining food preferences [35].

Most of the information provided in this study focused on attributes and consequences of bitter melon consumption that would benefit individuals at higher risk for diabetes or CVD. Although diabetes is considered as an old age disease, the prevalence of T2DM is increasing in children and young adults, especially in the Pacific region [36]. Therefore providing bitter melon-associated health information to younger population may be critical in prevention of diabetes. However, statistical analysis indicated that age did not influence the consumption intention in our study (data not shown).

Major strength of our study was identifying ingredients from western diet that are able to effectively mask the bitterness of functional food such as bitter melon. Although the order of samples was not randomized, this study limitation may not necessarily affect the taste of subsequent samples since water and saltine crackers were provided between dishes to cleanse their palates. However, the first food sample was randomized so that each dish would not be influenced by the perceived bitterness of the other dishes. This is exemplified by the fact that the participants at the soup station did not all dislike the chili (the last to be sampled by these participants). Randomizing the first dish was an effort to eliminate any potential cumulative effect on taste and therefore may be perceived as "structured randomization".

Overall, our study demonstrated that incorporating bitter melon in commonly consumed dishes increased acceptability among a mixed-ethnic and variable age group. However, previous exposure and knowledge of its health benefits had no effect on consumption intentions. Although our study was limited in the number of bitter melon-containing dishes that were tested and the age of the population, acceptability of foods with masked bitter taste merits future studies with large number of bitter melon-containing food dishes.

Bitter melon is routinely consumed as a part of many Asian diets and should therefore be expected to have minimal side effects. Well-designed clinical trials are also warranted to verify the dosage, safety, and pharmacokinetics of bitter melon. Results obtained from such studies will be significant since the information can help to develop nutritional regimens that can be incorporated into a daily diet. Developing such dietary alternatives to increase palatability and ultimately consumption of bitter tasting functional foods is expected to positively impact diabetes outcomes. Such intervention strategies would also be valuable to populations where conventional therapy is inaccessible and/or unaffordable, thereby improving diabetes care and management.

## Conclusions

Overall, sour flavor of tomato based recipes were able to mask the bitterness of bitter melon and these food dishes were considered acceptable by most participants. Taste rather than health information was a major determinant of willingness to consume bitter melon in future. The potential benefits associated with many bitter fruits and vegetables, such as bitter melon, may warrant the development of recipes to use in nutrition interventions aimed at increasing consumption of these bitter foods. Future studies are warranted to test the palatability of additional bitter melon containing food choices among participants of varying ages.

## Abbreviations

CVD: cardiovascular diseases; SOC: stages of changes; T2DM: type 2 diabetes mellitus.

## Competing interests

The authors declare that they have no competing interests.

## Authors' contributions

SSL conducted the experiments and designed the questionnaire and participated in developing ideas and writing the manuscript. VRN provided critical insights in conceptualization and development of this project and in the process of revising draft versions of the manuscript. DAD and ACS provided critical insights in the designing of questionnaire and reviewing the manuscript. JTF and his team performed the statistical analysis. PVN, developed ideas, designed experiments and questionnaire, secured funding and wrote the manuscript. All authors have read and approved the final manuscript.
