# Correction: strategies to improve palatability and increase consumption intentions for *Momordica charantia* (bitter melon): a vegetable commonly used for diabetes management

**DOI:** 10.1186/1475-2891-13-3

**Published:** 2014-01-10

**Authors:** Laura S Snee, Vivek R Nerurkar, Dian A Dooley, Jimmy T Efird, Anne C Shovic, Pratibha V Nerurkar

**Affiliations:** 1 Department of Human Nutrition, Food and Animal Sciences (HNFAS), College of Tropical Agriculture and Human Resources (CTAHR), University of Hawaii, Honolulu, HI, USA; 2 Department of Tropical Medicine, Medical Microbiology and Pharmacology, John A Burns School of Medicine, University of Hawaii, Honolulu, HI, USA; 3 Center for Health Disparities Research and Department of Public Health, Brody School of Medicine, Greenville, NC, USA; 4 Laboratory of Metabolic Disorders and Alternative Medicine, Department of Molecular Biosciences and Bioengineering (MBBE), CTAHR, University of Hawaii, Honolulu, HI, USA

## 

This erratum is being published to provide additional clarifications in the methods and discussion section [1]. Corrected version of the methods and additional discussion is included in the current version of our manuscript.

## Corrections to “Recipe development and preptions”

Approximately 50 g of uncooked bitter melon per one cup (250 ml) of raw ingredients was added to each dish, except curry dish. The curry dish does not have any other ingredients besides bitter melon and therefore contained approximately 61 g of bitter melon per one-half cup (125 ml). Each of the raw ingredients in these recipes (except the curry dish) were measured prior to cooking to ensure that the pre-cooked amounts of uncooked bitter melon equalled to 50 g per one cup of raw ingredients in the recipe. In order to obtain the correct proportions of uncooked bitter melon and raw ingredients, bitter melon was added last based on the total weight of the other raw ingredients, for e.g. if the amounts of raw ingredients was two cups, 100 g of uncooked bitter melon was added to the recipe prior to cooking. Chinese variety of bitter melon used in these recipes contains almost 90% water by weight (Islam et al., 2011, Functional Foods in Healths and Disease: 2:61–74). Similarly, other ingredients such as other vegetables and broth also contain water. Therefore the amount of bitter melon per cup of the cooked recipes may differ based on cooking time and water losses due to evaporation. Detailed recipes for all the dishes are now included as supplemental material.

## Additions to “Discussion”

The curry dish was used as a negative control for bitterness to test the overall hypothesis that cooking bitter melon with other ingredients would mask or reduce the bitter taste rather than just cooking bitter melon with only seasonings and spices. As expected, the curry dish was the most disliked for bitterness, as it contained only bitter melon and therefore practically twice the amount of bitter melon as compared to other recipes. However, bitterness of 50 g of bitter melon curry dish is expected to be same as 100 g of bitter melon curry dish since this dish does not contain any other ingredients except bitter melon and seasonings or spices. In contrast, although the soup, chili and the pasta sauce contained approximately 50 g of bitter melon per cup of the raw recipe ingredient, the tomato-containing bitter melon recipes were most “liked”.
